# Early Educational Interventions to Prevent Gender-Based Violence: A Systematic Review

**DOI:** 10.3390/healthcare11010142

**Published:** 2023-01-03

**Authors:** Lourdes Villardón-Gallego, Alba García-Cid, Ana Estévez, Rocío García-Carrión

**Affiliations:** 1 Department of Education, University of Deusto, 48007 Bilbao, Spain; 2 Department of Psychology, University of Deusto, 48007 Bilbao, Spain; 3 Ikerbasque, Basque Foundation for Science, Plaza Euskadi 5, 48009 Bilbao, Spain

**Keywords:** school intervention, gender-based violence, education, public health, scientific evidence, children

## Abstract

Background: Gender-based violence is a worldwide public health problem that is increasingly occurring at younger ages. This investigation aims to analyze effective interventions to prevent and to face gender-based violence beginning in early childhood in order to ensure quality education for all children through violence-free schools. Methods: This research has conducted a systematic review of interventions that have demonstrated a positive impact on the prevention and reduction of gender-based violence from early ages up to 12 years, inclusive, in schools. An extensive search in scientific databases (WoS, SCOPUS, ERIC, PsycINFO) was conducted from 2007 to 2022. Results: Thirteen articles were selected and analyzed in-depth to identify the success factors of these interventions, which (a) are integrated into the school curriculum; (b) promote active participation of students and community; (c) are based on scientific evidence; and (d) make relevant adaptations to a specific group and context. Conclusions: The programs analyzed have had a positive impact on raising awareness of gender violence, overcoming stereotypes, improving relationships in the classroom and reducing violent behavior, as well as empowering the most vulnerable people.

## 1. Introduction

Gender violence is a significant public health problem affecting millions of individuals around the world [1]. Children are some of the most vulnerable populations affected by gender violence. Consequently, research has been focusing lately on exploring the harmful consequences of gender-based violence (GBV hereinafter) can have on children, especially later in their lives [2,3,4,5]. Young children’s exposure to gender violence is associated with negative outcomes in their development and can have devastating effects throughout their lives [6,7,8].

The World Health Organization has reported in 2021 that 30% of women between 15 and 19 suffer or have suffered GBV in their sexual affective relationships [9]. Moreover, regarding the European Union Agency for Fundamental Rights [10], one in ten European women has been a victim of sexual violence, including both before the age of 15 and after the age of 15. Indeed, gender violence occurs more and more at early ages, and it happens everywhere, including schools [11]. 

Whereas school violence and bullying are identified and their effects on the physical and mental health of children have been extensively studied [12,13], their underlying causes include social and gender norms and broader contextual and structural factors that remain unexplored. Actually, according to UNESCO, in 2020 much of the school violence and bullying are related to gender [14]. Furthermore, school related gender-based violence affects millions of children, families and communities. It involves acts or threats of sexual, physical or psychological violence occurring in and around schools, perpetrated because of gender norms and stereotypes and enforced by unequal power dynamics [15]. 

However, in the school context, gender violence is reported only occasionally and is less identified as a major problem in childhood [16]. Therefore, the lack of awareness about gender-based violence in childhood and very early in life may hinder opportunities for its prevention and for an effective intervention. 

Schools are ideal settings for promoting gender-based violence and abuse prevention, providing knowledge for children to recognize and reduce risk behaviors [17]. Therefore, it is important to identify effective strategies to prevent gender-based violence in the early school years. Correspondingly, the aim of this research is to conduct a systematic review of interventions to prevent gender-based violence in childhood, specifically from 3 to 12 years old, inclusive, as a way to ensure quality education for all children through violence-free schools.

## 2. Materials and Methods

In order to achieve this objective, a systematic review was conducted. This methodological approach allows us to define the relevant concepts of a field of study, synthesize evidence, identify previously used methodologies and highlight gaps in the literature and future fields of study [18]. In this investigation, we have thoroughly reviewed the scientific literature and systematized the relevant knowledge about our object of study: the effective prevention of gender-based violence from an early age in schools. 

To carry out the review, we follow the PRISMA statement [19] and the checklist by Joanna Briggs Institute [20], in order to offer transparency, validity, replicability and updateability in this study.

### 2.1. Review Design and Search Strategy

The protocol for conducting a systematic review consists of the following: defining purpose of the study; narrowing the search strategy; literature search in the selected databases; screening according to inclusion and exclusion criteria; quality assessing of publication; gathering relevant information; synthesizing of studies; and writing up [21].

First, the research questions related to the objective of identifying successful interventions and programs in preventing and overcoming gender-based violence from early years were stated:

- What programs and interventions have been implemented in school settings to prevent and reduce gender-based violence?
- Have they been successful? What effects have they achieved?
- What are their main characteristics?

Secondly, the search strategy was established (see Table 1). The literature search was conducted between March and December 2022. It focused mainly on the search for scientific articles published in impact-indexed journals (Web of Science, SCOPUS), as well as other journals included in Education and Psychology databases (ERIC, PsychINFO).

Table 1 shows the keywords that guided the search, as well as the target population and the proposed categories. The authors conducted the search by combining the different keywords in English and Spanish and using the Boolean operators “OR”, “NOT”, “AND”. In addition, the following criteria were considered:

- Period: last 15 years (from 2007 to December 2022).
- Type of document: article, report.

### 2.2. Inclusion and Exclusion Criteria

Third, the inclusion and exclusion criteria were formulated, with the aim of incorporating only the literature relevant to the purpose of this study.

- Inclusion criteria (to be included, a publication has to meet all the inclusion criteria):
  1. Educational intervention from 3 to 12 years old inclusive.
  2. Intervention focused on preventing or reducing gender-based violent behavior.
  3. Interventions with impact/outcome evaluation.
- Exclusion criteria (meeting one of these criteria implies the publication is excluded):
  1. Intervention at school age above 12 years old or prior to infant stage (3 years old).
  2. Intervention outside the school setting.
  3. Intervention not referring explicitly to gender-related violence.

Afterwards, the selected studies were examined in detail considering aspects related to (a) the relevance of the study to the scope of the review and (b) methodological reliability aspects such as the appropriateness of the method and data collection, claims and evidence.

## 3. Results

### 3.1. Bibliographic Search 

**Figure 1:**
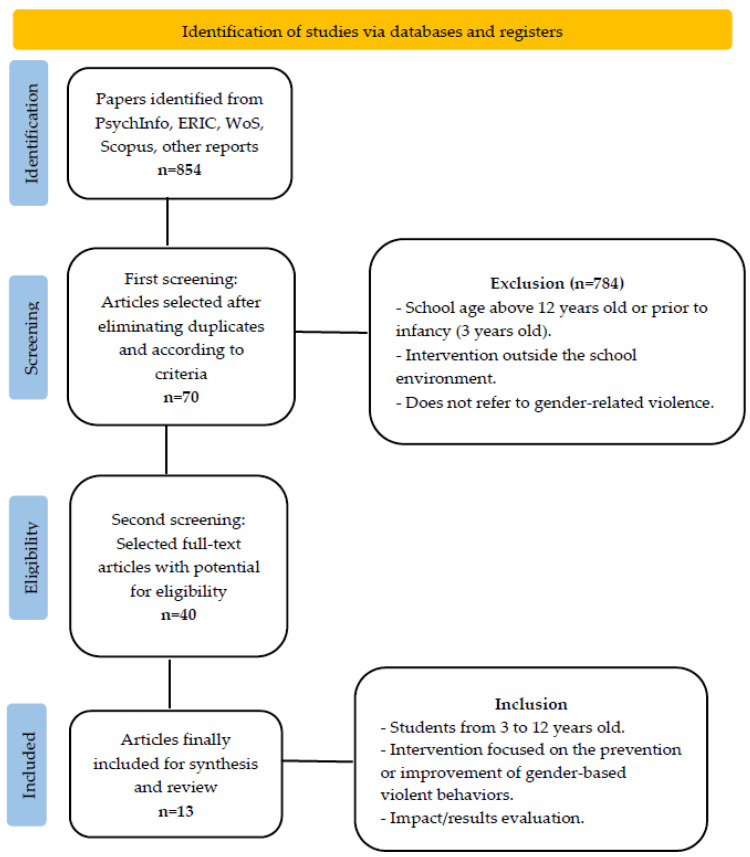
PRISMA flowchart.

Figure 1 shows the PRISMA flowchart where the whole bibliographic search process is outlined. A total of 854 records were retrieved through our searches in PsychInfo, ERIC, WoS and Scopus databases. After the identification phase, the first screening was carried out for further evaluation, where 70 articles were selected after eliminating duplicates and according to the inclusion criteria. Then, 40 papers where selected in the second screening, where the articles were analyzed and read full-text for eligibility. Finally, 13 studies were considered to be appropriate for the study.

An overview of studies’ main characteristics (country, type of study, method and target population) is showed in Table 2. 

Also, the quality of these studies was assessed using the Critical Appraisal Checklist for critical and interpretive research developed by the Joanna Briggs Institute [20]. The studies were checked against eleven questions. The results of the evaluation are presented in Table 3. It is worth mentioning that, in the articles by Devries et al. [22] and by McLaughlin et al. [23], one of the authors was also the designer of the educational intervention. However, no biases in the interpretation or in how the evaluation had been addressed have been noticed.

### 3.2. Characteristics of the Interventions

The interventions are described below according to the target population, objectives and methodology.

#### 3.2.1. Target Population

Eight interventions were focused on students, considering gender (one only for girls, one only for boys, six for both genders), while four interventions have included other agents as targets. 

On the one hand, Edwards et al. [27] evaluated the effectiveness of the *IMpower* program among American Indian girls; meanwhile, Banyard et al. [24] proposed the evaluation of a gender–transformative violence prevention program for middle school boys since the age of ten: The Reducing Sexism and Violence Program—Middle School Program (RSVP-MSP). On the other hand, several interventions were focused on both female and male students. This is the study reported by Kågesten et al. [29] who applied *IMPower* for girls and *YMOT* (Your Moment Of Truth) for boys in the East African context. Furthermore, Sarnquist et al. [31] applied *IMPower* for girls and Source of Strength for boys. Garzón and Carcedo [28] propose a prevention program for primary school children in Colombia; Shifting Boundaries program (SB) by Taylor and Mumford [33] is addressed to middle school students (sixth and seventh grades); Chung and Huang [25] and Doni [26] present intervention programs for preschoolers.

Five interventions include other participants as targets. For example, Devries et al.’s [22] program aims to reduce violence from school staff to students, as well as between peers (girls and boys). McLaughlin et al. [23] propose an intervention that includes the participation of several stakeholders apart from students, such as teachers, school heads, community stakeholders, parents and a resource person (from an NGO or education department). In the same vein, Smothers and Smothers [32] involve schools, community members and families in order to prevent sexual abuse. Ollis et al.’s intervention [30] includes three levels of professional development—school leadership, general staff and teaching staff—supporting schools to engage parents in the program. Moreover, schools were supported by experts from Senior Education Advisors. Dagadu et al.’s program [34], known as *GREAT* (Gender Roles, Equality and Transformations), includes male and female unmarried adolescents (10–14 years, 15–19 years), married adolescents (15–19 years) and adults (over the age of 19 years), using a stratified, two-stage cluster sample of primary and secondary schools and households.

#### 3.2.2. Objectives of the Interventions

Ten interventions are aimed at enhancing protective factors against gender-based violence, such as empowerment and sexual education. Three interventions focus on reducing violent behavior. 

Among those interventions aimed at promoting protective factors, the program evaluated by Edwards et al. [27] focuses on empowering young girls in areas such as verbal skills, physical resistance and extreme risk strategies. Kågesten et al. [29] aim to strengthen girls’ critical reflection and problem-solving skills and to boost their self-esteem and confidence, as well as to work into positive masculinities and skills for verbal bystander intervention in boys. The focus on Banyard et al. [24] is to increase positive expressions of masculinity, in order to work on their protective factors as a primary prevention strategy to reduce first time perpetration. Moreover, the intervention may serve as a secondary prevention to promote positive coping and reduce risk factors among students who are already at risk, that is, those students who have experienced victimization exposure prior to the prevention. Sarnquist et al.’s protocol [31] for girls was focused on empowerment, developing verbal skills and physical self-defense. The boys’ training was focused on promoting gender equality, developing positive masculinity and teaching safe and effective bystander intervention techniques. Furthermore, the main goal of the program published by Garzón and Carcedo [28] was to improve attitudes toward gender equality, decrease the acceptance of attitudes toward partner violence and develop socio-emotional competencies as a means of preventing gender-based partner violence. The program is divided into three didactic units: gender construction, gender-based partner violence and socioemotional skills.

On the other hand, McLaughlin et al.’s [23] study was centered on sex education, HIV and AIDS throughout pedagogical practices. Moreover, the intervention carried out in Ollis et al. [30] aimed to develop an awareness of positive and negative gender norms, reflect on one’s own identity and develop an understanding of gender-based violence as involving unfair/hurtful behaviors based on negative gender norms, and identify and practice respectful and gender-friendly behaviors. The study conducted by Doni [26] proposed to investigate preschool children’s gender preconceptions regarding professions and to establish, if they were triggered, to revise these preconceptions after their exposure to counter stereotypes. Along this line, Chung and Huang [25] aimed to determine whether exposure to counter-stereotypical information could break gender stereotypes in kindergarten children. Dagadu et al. [34] proposed a community-based program to promote gender-equitable attitudes and behaviors among children, adolescents and communities to reduce gender-based violence and improve reproductive health (SRH). This intervention was based primarily on the premise that gender identities established early in life shape the future path of boys and girls and that recognizing gender norms influences health-related behaviors, especially during adolescence when gender norms and identities begin to converge.

Furthermore, three interventions focus on reducing violent behavior, including The Good School Toolkit [22] that aims to reduce emotional violence, severe physical violence, sexual violence and injuries. Likewise, The Sexual Assault Primary Prevention Model with Diverse Urban Youth [32] was created to prevent sexual abuse, reducing tolerance of sexual violence and sexual harassment. In the same vein, the *SB* intervention [33] consisted of a primary intervention to prevent youth dating violence and sexual harassment when establishing young relationships in the near future.

#### 3.2.3. Strategies of Intervention

Apart from Doni’s intervention [26], which consists of a specific activity in two sessions, the rest of the programs are integrated into the school curriculum. 

Many of the programs are based on active learning, using techniques such as storytelling [24,34], role playing [23,24,31], radio drama and activity cards to each life stage [34], keeping a journal [23] and drawing on the experience of the community [23,34]. In the same vein, Smothers and Smothers [32] reported an intervention which is developed through role modelling and active behavioral skills training. 

Moreover, most programs use dialogue as a tool for learning and reflection to promote participation and co-creation. McLaughlin et al. [23] carry out an intergenerational dialogue to discuss complex socio-cultural problems and propose a toolkit that collects strategies for developing a co-constructed HIV and AIDS curriculum. Suggestion boxes are used to promote participation. The program by Devries et al. [22] includes activities such as dialogues, school assemblies, suggestion boxes, collective formulation of school policies, booklet clubs, student courts, etc. Similarly, Banyard et al. [24] employ peer-to-peer dialogue, while Smothers and Smothers [32] combined didactic instruction and discussion. Chung and Huang [25] promote discussion among students about several pictures. Furthermore, Doni’s intervention [26] consisted of involving participants in questions about gender suitability of each profession before and after viewing a documentary. Sarnquist et al.’s sessions [31] facilitated discussions, as well as verbal and physical skills practice. *GREAT* program [34] includes a toolkit of participatory activities in order to induce reflection, dialogue and action around gender inequitable attitudes, behaviors, SRH and GBV. In addition, health teams in each village were trained to improve access and quality of youth-friendly services

Finally, it is worth mentioning that of the 13 articles analyzed, 3 of them include the *IMpower* intervention as their main program. The intervention proposed by Edwards et al. [27] addresses the *IMpower* program among American Indian girls. Kågesten et al. [29] propose an intervention for girls (*IMPower*) and boys (*YMOT*) for the East African context. Finally, Sarnquist et al.’s protocol [31] was based on the *IMPower* program for girls’ intervention and the Source of Strength program for boys’ intervention. This program includes hands-on risk-reduction techniques for recognizing and resisting different forms of sexual harassment and violence, including boundary setting, diffusion tactics, verbal assertiveness or negotiation (e.g., name potentially threatening behaviors from abusers) and different forms of physical self-defense (e.g., bodily weapons) as the last resort. 

Some of the interventions required specific resources and equipment such as cameras to record learners’ participation during lessons [23], posters to increase DV/H awareness [33], picture-book stories [25], radio drama set and guides and activity cards [34], video recorded interviews [26] and multimedia resources [28,34].

### 3.3. Effects

The effects of the interventions have been analyzed under three categories: knowledge and awareness, empowerment, relationships and violent behavior.

#### 3.3.1. Knowledge and Awareness

The two studies in early childhood education [25,26] focus on interventions on gender stereotypes. Chung and Huang [25] concluded through an experimental study that cognitive-based intervention, such as a gender equality curriculum, break gender stereotypes in kindergarten children as assessed by the Picture Classification Task [35].

Doni [26] exposed kindergarten children to vocational counter stereotypes. Before intervention, children were asked about the gender suitability of certain professions: building worker, kindergarten teacher, police officer, pilot, lorry driver, astronaut, football referee and sea captain. After that, children were shown fragments of a video of interviews of real-life women in non-traditional gender professions situated in counter stereotypical work environments and talking about their professions. The activity generated statistically significant changes in children’s perceptions for half of the professions. The professions of the construction worker, lorry driver, football referee, sea captain, pilot and astronaut represent professional fields that were mostly associated with male individuals in both pre-video and post-video results.

After the implementation of the program by Ollis et al. [30] in early primary school students, boys and girls were significantly less likely to consider stereotypically masculine and feminine occupations and activities.

Edwards et al. [27] gauged the effectiveness of a sexual assault self-advocacy intervention of six classroom sessions with American Indian girls in grades 6–12. The results showed that girls in the intervention group significantly increased in all domains of self-defense knowledge. 

The program for gender-based intimate partner violence at primary schools in Colombia carried out by Garzón and Carcedo [28] proved to be effective in these variables: male and female gender stereotypes, gender stereotypes in romantic relationships, normative beliefs regarding strong aggression, weak aggression, aggression against women and men among themselves, affective empathy and attitudes about aggression in romantic relationships.

The socioecological model of sexual abuse prevention by Smothers and Smothers [32] was effective at increasing participants’ knowledge of sexual abuse, awareness of school and community support resources in the case of sexual assault and identification of features of healthy and unhealthy relationships. 

The intervention by Kågesten et al. [29] has enabled girls to recognize sexual assault. Moreover, boys have reinforced positive life values and gender-equal attitudes.

Regarding the *GREAT* program [34], several improvements in knowledge and attitudes related to gender inequity were found. Furthermore, it is important to point out the results of the evaluation conducted with a longitudinal cohort of boys and girls of 10–14 years shows that some of the initial effects of the intervention were maintained three years after and also that new positive SRH outcomes were obtained.

#### 3.3.2. Empowerment

The program analyzed by Edwards et al. [27] teaches girls skills to be used in a potential sexual assault situation and empowers girls to believe that they are worth defending. Empowerment can be reflected in the courage to dare to report assaults. Thus, in the study by Devries et al. [22], the number of cases of peer sexual violence in the last week and last term was low. However, the results suggest that the intervention was related to a borderline increase in the reporting of sexual violence for girls in particular (in both periods) although this did not reach statistical significance. With the strategy carried out by Kågesten et al. [29], the girls reported that they have increased their self-confidence. In the intervention by McLaughlin et al. [23] the children feel listened to, which increases their confidence to express their concerns and experiences, as well as their own leadership skills to become even community educators.

#### 3.3.3. Climate, Relationships and Violent Behavior

In the study by Edwards et al. [27], results showed that self-defense intervention increased significantly the efficacy of the participant girls to resist a sexual assault. In the quasi-experimental group, the incidence of sexual assault decreased by 80% and the incidence of sexual harassment decreased by 26%. In the study by Banyard et al. [24], middle school boys exposed to a healthy-masculinity-focused classroom curriculum showed decreased support for the use of violence in relationships.

According to the evaluation of the intervention put in place with The Good School Toolkit [22], a decrease in severe physical violence and injuries by school staff, emotional violence by school staff, emotional violence by peers and physical violence by peers was recorded.

In Kågesten et al.’s study [29], girls improved their ability to resist sexual assault through verbal and physical self-protective strategies, negotiate sexual consent and exercise agency. Boys increased their ability to avoid risky behaviors and “bad” peer groups and to understand and respect consent. 

In the study by Sarnquist et al. [31], the incidence of self-reported sexual assault among girls decreased, and boys reported significantly higher rates of bystander intervention to prevent sexual assault. The intervention by Taylor and Mumford [33] was associated with significant reductions in the frequency of sexual harassment perpetration and victimization, the prevalence and frequency of sexual dating violence victimization and the frequency of total dating violence victimization and perpetration. In the study by McLaughlin et al. [23], adults, such as teachers themselves, overcome the myths and perceptions of children as innocent, passive and irresponsible. Through dialogue, different attitudes and beliefs are expressed in an atmosphere of respect, where participants listen to each other and value other points of view. Furthermore, after the intervention, the teaching methodology changed from being mainly teacher-centered to promoting student participation.

The *GREAT* program’s results were also promising [34]; a significant improvement in behaviors related to gender inequity were observed. Specifically, seven of the nine life-stage measures showed significant changes toward greater gender equity. For example, a significant increase was observed in the number of brothers helping their sisters with household chores and in the number of brothers talking to their parents about their sisters’ education.

### 3.4. Success Factors

After analyzing the main characteristics of the interventions, as well as their effects, several success factors have been identified. These factors are recurrently present in different interventions with a positive impact improving the identification, prevention or reduction of gender-based violence in the school context. 

#### 3.4.1. The Intervention Is Integrated into the School Curriculum

One of the recurring factors identified in the analyzed programs is to integrate the intervention into the school curriculum so that it is a part of the students’ academic activities more than a one-time or sporadic activity. Thus, curriculum interventions are found in 12 of the 13 proposals [22,23,24,25,27,28,29,30,31,32,33,34].

As with academic performance, active learning is a key factor in the success of the programs analyzed [23,24,31,32,34].

#### 3.4.2. Student Participation, Dialogue and Co-Creation

Another aspect frequently found in interventions is to consider the students themselves when designing activities and plans for the prevention and reduction of gender-based violence. Their active participation, listening to their voices, priorities and languages, can be found in Devries et al. [22], McLaughlin et al. [23], Chung and Huang [25], Garzón and Carcedo [28], Kågesten et al. [29], Ollis et al. [30], Sarnquist et al. [31] and Dagadu et al. [34].

#### 3.4.3. Safe School Environment

In order to encourage participation and dialogue on these issues, it is important that the school is a safe environment where supportive social relationships take place. For example, Smothers and Smothers‘ program [32] relied mainly on two important aspects: (a) the calculated placement of sexual abuse prevention within the context of a healthy relationship curriculum and (b) the importance of building healthy relationships between community agencies, school professionals, students, and parents/guardians. A safe school environment may neutralize any shame-based avoidance of discussing sensitive topics. Ollis et al.’s intervention [30] is based on establishing respectful and egalitarian relationships. Similarly, McLaughlin et al.’s program [23] is based on creating safe environments through participation and dialogue.

#### 3.4.4. Considering Scientific Evidence of Successful Programs and Rigorous Models

The programs included in this systematic review have been developed according to theoretical models about how to prevent gender-based violence and, also, according to strategies that have already demonstrated their effectiveness in the past [22,23,24,25,26,28,29,30,31,32,33,34]. 

#### 3.4.5. Involving Relevant Community Agents

Important agents for the development of children, such as family, parents and tutors [23,29,32,34]; the community [23,27,31,34]; as well as experts [23,30,31]; victims of gender-based violence [29]; or health workers [34], have been taken into account in several interventions, in addition to teachers [29,31], head teachers and school leaders [30,31] and even politicians [31].

#### 3.4.6. Adapting the Intervention to the Specific Target and School Context

Finally, several interventions explicitly state that they have adapted the intervention content, approach or methodology according to the specific target audience or context [23,24,25,26,27,29,30,31,32,34]. 

## 4. Discussion

The aim of this research is to carry out a systematic review of gender-based violence prevention and intervention programs implemented in the early educational stages. Considering the inclusion and exclusion criteria, thirteen articles have been selected: two applied in pre-school education (from 3 to 6 years old) and eleven describing programs developed with students from 6 to 12 years old, inclusive. 

The results confirm that there are few interventions aimed at this subject in these school stages. One of the reasons that may explain this scarcity is that teachers and other adults do not interpret various behaviors related to gender-based violence as gender-based violence. In fact, the problem of GBV begins to be considered from adolescence or late childhood onwards [35,36,37], when the number of interventions increases considerably. In fact, the only two interventions that have been located in preschool children are aimed exclusively at addressing gender stereotypes [25,26].

However, considering the negative consequences that such violent experiences have on development, it is important to investigate effective prevention and intervention programs. The interventions identified in this review have been shown to be effective in overcoming gender stereotypes. Research has shown how stereotypes hinder gender equality and can encourage violent behavior [38,39,40]. Therefore, raising awareness against gender stereotypes is a step in the prevention of gender-based violence. Overcoming stereotypes is related to increasing knowledge about the topic and raising awareness among students, which is the result of several of the research studies analyzed [25,26,27,28,29,30,32]. Research has identified vulnerability to violence in people with low self-esteem [41] and insecurity [42]. Consequently, empowerment should be an important goal of violence prevention programs [43]. 

Furthermore, several of the studied programs to prevent gender-based violence have an effect on improving relationships and school climate [23,32]. Indeed, a safe school environment supports optimal development and effective learning [44].

Most of the studied programs are integrated into the curriculum, which facilitates their success in the school context [45]. Moreover, the participation of other members of the community is related to learning effectiveness [46]. 

A feature shared by several successful programs is the promotion of dialogue and student participation [22,23,25,28,29,30,31]. Research has shown the importance of listening to the voices of students to create egalitarian and safe contexts [47,48]. 

It is essential to base programs on scientific evidence, i.e., on other interventions that have shown to be effective in preventing gender-based violence. Evidence-based education enables a quality education for all students [49,50]. Obviously, effective interventions need to be transferred and adapted to the specific context and target population [51], as several of the analyzed articles have considered [23,25,26,27,29,30,31,32].

After all, it is necessary to point out that this systematic review has had several limitations. The bibliographic research has been carried out in articles published in English and Spanish. Likewise, the search was conducted exclusively in databases of scientific publications and reports of international organizations. Consequently, there may be other programs that could have been considered for this investigation. In the same way, the thirteen studies analyzed correspond to different and specific cultural contexts. Culture has a fundamental relevance in explaining the behavior of the people who are part of it. Cultural ideologies can both empower and oppress women, and, therefore, culture has the potential to increase the risk of experiencing gender violence and associated outcomes or protect against these factors [52]. Therefore, it is important to consider how cultural factors can influence disparities in both the nature of interpersonal violence that is experienced and the potential outcomes [53]. Furthermore, another issue that has arisen is that education systems vary from country to country so that the same age range could correspond to primary, middle or secondary school.

For all the above mentioned, it is proposed that, in future investigations, factors—such as different languages, cultures and education systems—should be addressed when analyzing the intervention and prevention programs. It would also be essential to scientifically analyze the causes of gender violence in the fight to eradicate this social scourge.

## 5. Conclusions

This systematic review has made it possible to analyze effective programs in the prevention and intervention against gender-based violence from early educational stages. Likewise, the analysis of the selected articles has led to the identification of important success factors to take into consideration when designing or planning actions aimed at preventing gender-based violence at school—such as to be integrated in the curriculum, to promote students’ activity, to dialogue and to participate in a safe and supportive environment, to encourage the participation of the families and community members, to be based on scientific evidence and to adapt the intervention to target and context. These findings should lay the groundwork for the creation of public policies for equality and the eradication of gender-based violence from an early age.

## Figures and Tables

**Table 1:** Search strategy.

Authors	Keywords		
	Target and Database	Prevention/Intervention	Population/Context
1	Early Childhood (under 6 years old) Kindergarten Pre-school	Gender/sex-based violence Sexual harassment Gender/sex bullying Gender/sex inequality Gender/sex disrespect Gender/sex discrimination Gender violence Gender equality Strategies Programs Intervention Prevention	Education Children Student Classroom School Pupil Teachers Children’/Students’ Families
2 y 3	Elementary Primary School age (from 6 to 12 years old)		

**Table 2:** Overview of the studies’ main characteristics.

Source	Country	Type of Study	Method	Target Population
Devries et al. [22]	Uganda	Experimental	Survey	5th–7th g. (11–14 y.)
McLaughlin et al. [23]	Botswana, Ghana, Kenia, Sudáfrica, Swaziland, Tanzania	Qualitative/Action research	Observation, semi-structured interviews, Focus-group, dialogue of all stakeholders	Primary school
Banyard et al. [24]	USA	Quasi-experimental	Survey	Middle school (6th–8th g.) (10–15 y.)
Chung and Huang [25]	Taiwan	Experimental	Picture Classification Task	Preschool (5–6 y.)
Doni [26]	Grece	Pre/Post	Profession gender-based images Task	Preschool (4–6 y.)
Edwards et al. [27]	USA	Quasi-experimental	Survey	Middle and High school girls (6th–12th g.) (12–18 y.)
Garzón and Carcedo [28]	Colombia	Quasi-experimental	Survey	Primary school (2th–3th g.) (7–9 y.)
Kågesten et al. [29]	Kenia	Qualitative	Focus Groups, In-Depth Interviews	Students (10–19 y.)
Ollis et al. [30]	Australia	Pre/Post	Survey	Primary school
Sarnquist et al. [31]	Kenia	Two-arm, parallel, cluster-randomized trial	Survey, In-deth interviews	10–14 y.
Smothers and Smothers [32]	USA	Quasi-experimental	Survey	5th–12th g.
Taylor and Mumford [33]	USA	Multi-level experimental design	Survey	Middle School (6th–7th g.) (12–13 y.)
Dagadu et al. [34]	Uganda	Repeated Cross-sectional evaluation study	Survey	From school (10–19 y.) to adulthood

**Table 3:** Quality of studies.

Source	Q1	Q2	Q3	Q4	Q5	Q6	Q7	Q8	Q9	Q10
Devries et al. [22]	Yes	Yes	Yes	Yes	Yes	Yes	No	Yes	Yes	Yes
McLaughlin et al. [23]	Yes	Yes	Yes	Yes	Yes	Yes	No	Yes	Yes	Yes
Banyard et al. [24]	Yes	Yes	Yes	Yes	Yes	No	No	Yes	Yes	Yes
Chung and Huang [25]	Yes	Yes	Yes	Yes	Yes	No	No	Yes	Yes	Yes
Doni [26]	Yes	Yes	Yes	Yes	Yes	No	No	Yes	Yes	Yes
Edwards et al. [27]	Yes	Yes	Yes	Yes	Yes	No	No	Yes	Yes	Yes
Garzón and Carcedo [28]	Yes	Yes	Yes	Yes	Yes	No	No	Yes	Yes	Yes
Kågesten et al. [29]	Yes	Yes	Yes	Yes	Yes	No	No	Yes	Yes	Yes
Ollis et al. [30]	Yes	Yes	Yes	Yes	Yes	No	No	Yes	Yes	Yes
Sarnquist et al. [31]	Yes	Yes	Yes	Yes	Yes	No	No	Yes	Yes	Yes
Smothers and Smothers [32]	Yes	Yes	Yes	Yes	Yes	No	No	Yes	Yes	Yes
Taylor and Mumford [33]	Yes	Yes	Yes	Yes	Yes	No	No	Yes	Yes	Yes
Dagadu et al. [34]	Yes	Yes	Yes	Yes	Yes	No	No	Yes	Yes	Yes
Q1. Is there congruity between the stated philosophical perspective and the research methodology? Q2. Is there congruity between the research methodology and the research question or objectives? Q3. Is there congruity between the research methodology and the methods used to collect data? Q4. Is there congruity between the research methodology and the representation and analysis of data? Q5. Is there congruity between the research methodology and the interpretation of results? Q6. Is there a statement locating the researcher culturally or theoretically? Q7. Is the influence of the researcher on their search, and vice-versa, addressed? Q8. Are participants, and their voices, adequately represented? Q9. Is the research ethical according to current criteria or, for recent studies, and is there evidence of ethical approval by an appropriate body? Q10. Do the conclusions drawn in the research report flow from the analysis or interpretation, of the data?										

## Data Availability

Not applicable.
